# Quick Control of Pain and Disease without Drugs by Use of the Leucodescent Light

**Published:** 1908-07-15

**Authors:** F. E. Raiche

**Affiliations:** Marinette, Wis.


### QUICK CONTROL OF PAIN AND DISEASE WITHOUT DRUGS BY USE OF THE LEUCODESCENT LIGHT.

BY F. E. RAICHE, D. D. S., MARINETTE, WIS.

I believe I come before you with something comparatively new in dental therapeutics.

I had read a few times of how pain was quickly controlled and pus formation checked by the application of electric light, such as blue light, Finsen rays, combined rays or leucodescent, etc. I then read up all I could find on light therapeutics, including the X-rays, and decided to try the leucodescent, as it is free from ill effects, such as burning, which is a decided point against the X-rays, and I dropped the blue light because it was not as powerfully germicidal as the leucodescent.

Several authorities claim that it is the blue, violet and ultra-violet rays in the sunlight, and not the heat rays, that have a destructive action upon putrefactive bacteria. This range in the light spectrum is fully covered by the leucodescent light. These are powerfully germicidal, destroying the various pus germs and practically all other pathogenic bacteria, without injury to normal tissue cells.

The violet rays are also powerfully anodyne, and in no way injurious, as is the case with narcotics.

In neuralgias and acute inflammations, the pain-relieving action of the leucodescent light is one of its most valuable qualities. Inflammatory conditions, local or deep seated, can be nicely controlled, because the rays relieve congestion and prevent stasis by restoring equilibrium and toning up the tissues.

The *Dental Summary* of September, 1904, contains an article by Dr. Arthur E. Peck of Minneapolis on the use of this light, in which he says he cured headache for a patient in seven minutes, and a severe neuralgia of his own in seven minutes. He treated pyorrhea successfully, stopping suppuration in a few treatments and reduced the swelling and pain from extraction infection with the light only. It was this article that caused me to decide on the leucodescent lamp.

At just about the time I was studying on the subject I got an epidemic of severe cases of suffering and ordered the lamp on short notice and here is the way I used it:

The first case was an impacted third molar, causing suppuration on the buccal side between the second and thirdmolars. Jaw was largely swollen, teeth closed and set, pain intense. Had not been able to open mouth wide enough for solid food for a week. Physician could not relieve pain with the usual narcotics. He was sent to me to extract the tooth if I could open the jaw. I placed him under the light twenty minutes, which relieved him entirely of pain, and the hard lump on jaw softened. After three days' treatment twice a day he could open his mouth to normal. I opened abscess, and with continued light treatments and washings I cured the case without extracting the tooth and his suffering ceased after first application of the light.

Case 2. A blind abscess over upper central, face swollen and aching severely. I anesthetized with gas and lanced gum and opened tooth. After he awoke I washed through the canal, which gave him great pain. A few minutes under the light relieved him. Upon exploring this canal I found my broach readily reached about three-quarters of an inch beyond the root, and when I pushed in the lance it dropped into space above the tooth. My only treatment after opening was washing a few times with glyco-thymoline and light treatments daily for a week, then every other day for a week, and weekly for a month, when I discharged him. This surely was a good test case, as remember my only treatment was washing and the light. I purposely avoided using cauterizing, drugs or opening to cut away dead bone, depending on the light to assist Nature to take care of it.

Case 3. Blind abscess over upper lateral incisor, a sound tooth and very sore to touch. Placed under light and after five minutes' exposure was able to drill through tooth without pain. This shows the powerful anodyne effect and this is where I very often use it. It is well worth having if it will enable us to work on sore teeth without pain.

Case 4. In one severe case of pyorrhoea a swelling developed midway between the lower second and third molars. I opened and curetted a little, scaled the roots of all teeth affected, there being about a dozen, washed the pockets and then used only the light and glyco-thymoline daily on the start, gradually diminishing treatments until after about a month, when the mouth looked fine.

That patient told others that I did wonders for him. Since I gave him that treatment about a year and a half ago he has dropped in every few months to get a light bath to keep the gums in good condition. Last winter when I gavehim a treatment he had about a dozen boils on his neck. I applied the light to those twice and they disappeared. Last week I cleaned his teeth and found not a sign of pyorrhea.

I have cured many cases of severe pain and stiff muscles from erupting third molars with just a few applications of the light. Some of these were accompanied by suppuration caused by the pressure. I lanced and washed them, injected a drop of saturate solution of camphophenique and healed them with the light.

A few weeks ago a young lady came to me complaining of soreness and stiffness of the muscles of her jaw on one side of a few weeks' duration. Three daily applications of the light entirely relieved her.

Last summer I placed an orthodontia apparatus on a girl's teeth and the next day she presented the worst case of pericimentitis I ever want to see. She begged me to remove it at once. Three light treatments that day relieved her and I used it daily until the tissues became accustomed to the pressure and I had no further trouble with her. I know that if I had not used the light I would have had to drop the case.

Whenever a patient complains of a headache while in the chair I relieve them with the light.

Last winter I was troubled very much with cold feet and legs as far up as my knees, caused by poor circulation. One day I had to dismiss a few patients because I couldn't stand any longer at the chair, and while I was walking up and down the floor the thought occurred to me that the light would warm them up and restore circulation, so I tried it and sure enough, two treatments warmed them up for good.

Here is a case I would like to know how you would relieve so quickly if at all. A dentist down the street injected cocaine and extracted two roots from an otherwise edentulous jaw. Next day the patient returned with a sore jaw. The dentist injected dioxygen and put in a cotton plug and left it. Of course, it grew worse. The man went to a physician, who gave him morphine and a dilute glyco-thymoline wash. It grew worse and the morphine didn't help matters. The physician sent him to me a week from the start of the trouble. The man told me he hadn't slept for a week, and the day before he became so crazed with pain he smashed part of the furniture in the house. Fifteen minutes after he arrived in my office he was entirely free from pain and the light did it. A large area of his jaw was honeycombed and full of pus. I curretted it, washed it and used the light to cure it. I considered it a fit case for the surgeon's knife, but avoided it.

Here is another interesting case which came to me a few weeks ago. A young man arrived from the country with a complete fracture of the lower jaw at the angle through the alveoli of the third molar, the tooth being retained in the ramus half, the first and second molars being previously missing. He said he had been done up by five others three days previous. He supposed the molar should be extracted because it was loose, not realizing that it was his jaw-fragment that was loose. His whole face and neck was battered and swollen and black and blue. I used the light on him three times within two hours, this relieving the pain and soreness almost entirely, enabling me to handle the jaw without pain. I then reduced the fracture, secured the parts, gave him another light bath and let him go. He said the light made the jaw feel good. He returned two days later in a perfectly normal condition. The swelling, discoloration and pain having entirely disappeared. The only reason for that quick resolution that I can give is the use of the light.

In the line of neuralgia the most important case I had was in the supra-orbital nerves. The attacks came on every day and lasted from three to six hours. A five-minute exposure completely stopped the pain every time. A month's daily treatment cured the case.

Whenever it is necessary to use drugs the auxiliary application of the light will hasten its action.

"The light and heat rays cause pronounced dilatation of superficial blood vessels, relieving congestion, stimulating circulation, and promote absorption."

"The pronounced counterirritant effect, coupled with dilatation of superficial vessels and relief of congestion of the parts produce analgesic action which is gratifying.

The light is made up in 300, 400 and 500 candlepower intensities, all having the same effect, the higher ones giving a quicker and more penetrating effect.—*Review.*

